# The Peroxisomal Targeting Signal 1 in sterol carrier protein 2 is autonomous and essential for receptor recognition

**DOI:** 10.1186/1471-2091-12-12

**Published:** 2011-03-04

**Authors:** Chris P Williams, Nicole Schueller, Colin A Thompson, Marlene van den Berg, Simon D Van Haren, Ralf Erdmann, Charles S Bond, Ben Distel, Wolfgang Schliebs, Matthias Wilmanns, Will A Stanley

**Affiliations:** 1 EMBL-Hamburg, c/o DESY, Notkestraβe 85, 22603 Hamburg, Germany; 2 Department of Medical Biochemistry, Academic Medical Center, University of Amsterdam, Meibergdreef 15, 1105 AZ Amsterdam, The Netherlands; 3 Phylogica, 100 Roberts Road, Subiaco, 6008 WA, Australia; 4 Department of Systems Biochemistry, Institute for Physiological Chemistry, Faculty of Medicine, Ruhr University of Bochum, 44780 Bochum, Germany; 5 School of Biomedical, Biomolecular and Chemical Sciences, MCS Building (M310), University of Western Australia, 35 Stirling Highway, Crawley, 6009 WA, Australia; 6 ARC CoE in Plant Energy Biology, MCS Building (M316), University of Western Australia, 35 Stirling Highway, Crawley, 6009 WA, Australia

## Abstract

**Background:**

The majority of peroxisomal matrix proteins destined for translocation into the peroxisomal lumen are recognised *via *a C-terminal Peroxisomal Target Signal type 1 by the cycling receptor Pex5p. The only structure to date of Pex5p in complex with a cargo protein is that of the C-terminal cargo-binding domain of the receptor with sterol carrier protein 2, a small, model peroxisomal protein. In this study, we have tested the contribution of a second, ancillary receptor-cargo binding site, which was found in addition to the characterised Peroxisomal Target Signal type 1.

**Results:**

To investigate the function of this secondary interface we have mutated two key residues from the ancillary binding site and analyzed the level of binding first by a yeast-two-hybrid assay, followed by quantitative measurement of the binding affinity and kinetics of purified protein components and finally, by *in vivo *measurements, to determine translocation capability. While a moderate but significant reduction of the interaction was found in binding assays, we were not able to measure any significant defects *in vivo*.

**Conclusions:**

Our data therefore suggest that at least in the case of sterol carrier protein 2 the contribution of the second binding site is not essential for peroxisomal import. At this stage, however, we cannot rule out that other cargo proteins may require this ancillary binding site.

## Background

**Figure 1:**
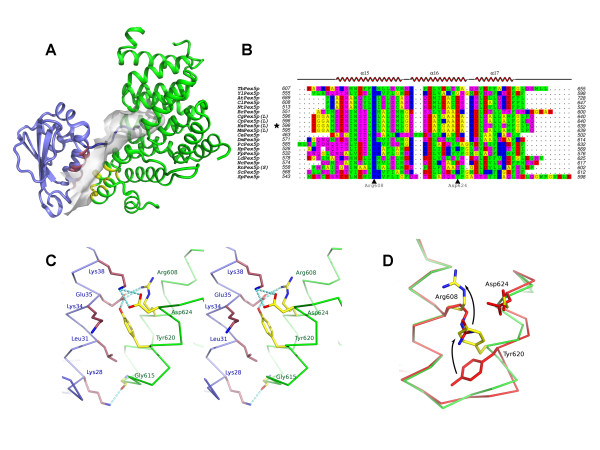
**Structural features of the Pex5p-SCP2 ancillary interface**. **(A) **Cartoon of the X-ray crystal structure of Pex5p(C) in complex with mSCP2 [4]. Pex5p(C) is shown in green and mSCP2 in blue. A partial surface representation for mSCP2 is shown, denoting the residues of mSCP2 that approach Pex5p(C) to < 4 Å - that is, the extended C-terminal PTS1 buried within Pex5p(C) and the ancillary helix-helix interface. The ancillary interface is highlighted - residues contributed by mSCP2 are coloured in burgundy and those contributed by Pex5p(C) in yellow. **(B) **Amino acid sequence alignment of the C-terminal 3-helix segment of Pex5p, using the standard letter code. The following colour scheme is used: CFILMPVWY (green), HNQST (magenta), DE (red), KR (blue) and AG (yellow). The alignment was generated with CLUSTAL-W [21] and manually adjusted, the figure generated in ALINE [22]. Positions of α-helices 15, 16 and 17 (from the human Pex5p(C)/mSCP2 crystal structure, PDB accession 2C0L, [4]) as defined by DSSP [23] are indicated at the top of the figure. Residues marked with an arrowhead (black triangle) were mutated in this study. Species identifiers: *Tb, Trypanosoma brucei; Yl, Yarrowia lipolytica; At, Arabidopsis thaliana; Cl, Citrullus lanatus; Nt, Nicotiana tabacum; Br, Brachydanio rerio; Cg, Cricetulus griseus; Cp, Cavia porcellus; Hs, Homo sapiens; Mm, Mus musculus; Ce, Caenorhabditis elegans; Dm, Drosophila melanogaster; Pc, Penicillium crysogenum; Hp, Hansenula polymorpha; Pp, Pichia pastoris; Ld, Leishmania donovani; Nc, Neurospora crassa; Rn, Rattus novegicus; Sc, Saccharomyces cerevisiae; Sp, Schizosaccharomyces pombe*. The human Pex5p sequence is indicated with an asterisk (black star). In mammalian sequences, (L) and (S) denote whether the numbering used refers to the long or short forms of Pex5p. **(C) **Stereo view focusing on the ancillary interface between Pex5p(C) and mSCP2 [4]. The protein colour scheme is as in **(A)**. Sidechains are shown for residues at the interface. See text for details. **(D) **View showing the different arrangement of key Pex5p residues at the ancillary interface in the presence (green) and absence (red) of mSCP2 [4]. See text for details. Crystal structure figures were prepared in PyMOL (http://www.pymol.org).

Pex5p, the major import receptor for peroxisomal matrix proteins, is known to carry folded proteins across the peroxisomal membrane by a signal assembled shuttling mechanism [1,2]. It has been found to recognise the type 1 Peroxisomal Targeting Signal (PTS1) - a C-terminal tripeptide of consensus sequence -[S/A/C]-[K/H/R]-[L/M]-CO_2_^-^, carried by some 40 human proteins destined for the peroxisomal lumen. The PTS1 sequence binds to Pex5p in an extended conformation, which is accommodated in a deep cavity in the tetratricopeptide repeat (TPR) domain of Pex5p [3,4] (Figure 1A). An additional interface, remote from the PTS1 binding site, of about 500 Å^2 ^has been found to form between Pex5p and the model cargo protein sterol carrier protein 2 (SCP2), as demonstrated by the crystal structure and solution studies of SCP2 in complex with the C-terminal region of Pex5p [4]. In the following we will refer to the interface as "ancillary" or "secondary". This interface utilises the first and fourth α-helices of SCP2 and helices 15 and 16 from the Pex5p C-terminal helical bundle (Figure 1A & 1B) and its formation confers an increased binding affinity of around 6-fold when compared to a minimal PTS1 hexapeptide (PGNAKL-CO_2_^-^), derived from the C-terminus of SCP2 [4]. Taken together, these observations may imply the presence of an additional receptor recognition element in SCP2, supplemental to the PTS1.

Inspection of the secondary interface of the Pex5p(C)-SCP2 complex structure reveals several intermolecular hydrogen bonds (Figure 1C), in particular: the carboxylate group of Glu35 from SCP2 interacts with the side chains of two Pex5p residues, Arg608 and Tyr620; Lys38 from SCP2 forms a salt bridge with Asp624 from Pex5p; finally, Lys28 from SCP2 interacts with the main chain carbonyl group of Gly615 from Pex5p(C). Comparison with the Pex5p(C) apo-structure [4] reveals that in the absence of SCP2, two residues from this interface, Arg608 and Asp624, re-orient their sidechains to form an intramolecular salt bridge, inducing a re-orientation of the sidechain of Tyr620 (Figure 1D). An alignment of a set of Pex5p sequences (Figure 1B) indicates that while Tyr620 and Asp624 are only partly conserved, a basic residue at the position equivalent to Arg608 in human Pex5p is invariant.

In this study, we have made point mutations of two key residues of the ancillary SCP2 binding interface in Pex5p, Arg608 and Asp624, to tryptophans to assess the relative importance of the additional interface in the recognition of SCP2 by Pex5p. To dissect the contributions of the PTS1 and these additional interactions, we also used a version of SCP2 lacking the C-terminal PTS1, for comparison. By four different approaches we demonstrate that while receptor-cargo binding and SCP2 transfer into the peroxisome are absolutely dependent on the presence of the PTS1, the ancillary interface is found to be dispensable - mutations effecting binding do not inhibit peroxisomal import *in vivo*. These results indicate that the ancillary interface is unlikely to play an important role in the import of the PTS1 protein SCP2.

## Results

### Yeast two-hybrid analysis of human Pex5p interactions with SCP2

**Figure 2:**
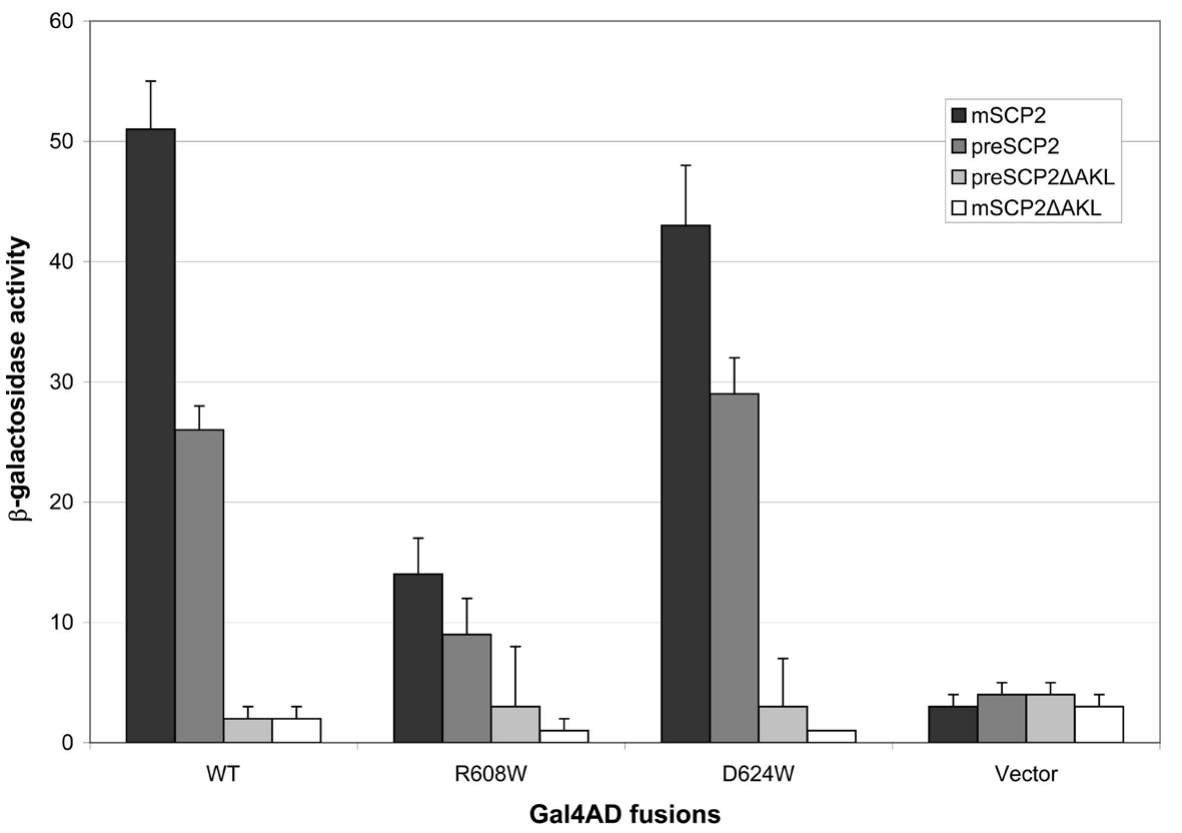
**Summary of yeast two-hybrid data**. Two-hybrid interactions of wild-type (WT) and mutant (R608W and D624W) forms of Pex5p(C) with SCP2. Activity of the reported gene β-galactosidase (as defined by absorbance at 420 nm per mg of protein per min) was used to determine the strength of interaction. Values correspond to the mean ± SD of three independent measurements.

**Figure 3:**
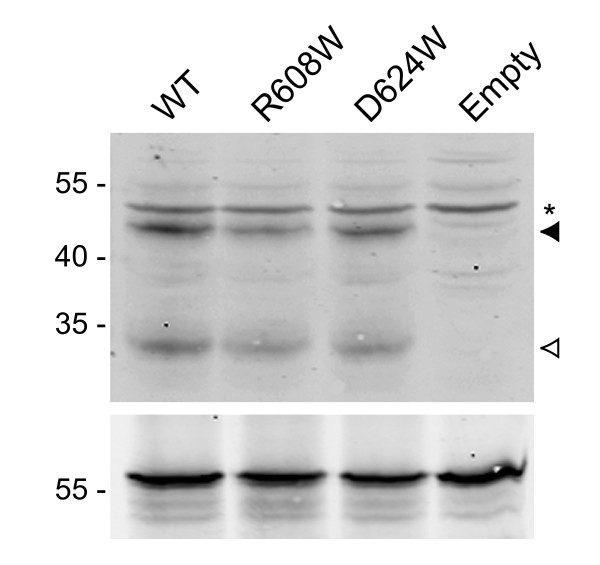
**Western blot showing Pex5p wild-type and mutant expression levels**. *Upper panel *- Expression levels of wild-type (WT) and mutant (R608W and D624W) forms of Pex5p in yeast cells used for two-hybrid analysis. The Gal4AD-Pex5p(C) fusions are indicated with a solid arrow. The open arrow indicates a breakdown product of the fusions and the asterisk indicates a background band recognised by the Pex5p antibody. *Lower panel *- Loading control, showing the same samples above probed with the anti-hexokinase antibody.

First, we created mutant versions of the cargo-binding domain of Pex5p (Pex5p(C), consisting of residues 315-639) and tested their capacity to interact with different versions of SCP2 in the yeast two-hybrid system (Figure 2). As a control, we showed that the presence of the PTS1 of SCP2 is a pre-requisite for binding to Pex5p(C), as no interaction could be detected in its absence, thus confirming previous findings [4]. The introduction of the mutation R608W into Pex5p(C) did result in a three-fold reduction of the interaction with both pre- and mSCP2. In contrast, the Pex5p(C) D624W mutation showed little affect, either leading to a slight decrease (mSCP2) or increase (preSCP2) of the interaction. To confirm that the observed decrease with the Pex5p(C) R608W construct was not due to problems with expression or stability of the protein, we performed Western blotting analysis with Pex5p antibodies on yeast lysates from cells expressing the different Pex5p(C) variants. We could see that all Pex5p constructs were expressed at similar levels (Figure 3).

### In vitro Pex5p-SCP2 binding

We tested the capacity of these mutants to bind mSCP2 *in vitro *using isothermal titration microcalorimetry (ITC) (Table 1). We observed that the Pex5p(C) R608W mutation resulted in a two fold reduction in SCP2 binding, in agreement with our yeast-two-hybrid data. In addition, we found the binding stoichiometry reduced to 0.61, indicating that only about two-thirds of the receptor was competent for SCP2 cargo binding, compared to a value approaching one for wild-type. The binding stoichiometry was further lowered to 0.41 when using the second Pex5p(C) mutant, D624W, coupled with an apparent tighter binding when compared to wild-type Pex5p(C). Additionally, we measured the binding affinity of the Pex5p(C) mutants against a peptide derived from the C-terminus of SCP2 (Table 1, PGNAKL [4]), a binding event that is independent of the ancillary interface. The Pex5p(C) R608W mutant bound to the peptide with a similar affinity as the wild-type protein. Furthermore, the binding stoichiometry of this mutant was closer to that of wild-type Pex5p(C), suggesting that the R608W mutant is impaired in binding when utilising the secondary interface but not when using the PTS1 binding site alone.

**Table 1:** Summary of ITC data

**Pex5p(C)**:	Ligand	n	ΔH (kJ/mol)	TΔS (kJ/mol)	ΔG (kJ/mol)	K_d _ (nM)
**Wild-type (5) ^a^**	mSCP2	0.93 ± 0.03	-43.4	-1.8	-42.3	95 ± 21

**R608W (4)**	mSCP2	0.61 ± 0.06	-71.7	-31.8	-39.9	179 ± 19

**D624W (4)**	mSCP2	0.41 ± 0.08	-55.6	-12.2	-43.4	45 ± 8

**Wild-type (2)**	PGNAKL	0.94 ± 0.02	-42.5	-5.6	-36.9	547 ± 6

**R608W (2)**	PGNAKL	0.81 ± 0.01	-51.6	-15.1	-36.5	635 ± 25

**D624W (2)**	PGNAKL	0.49 ± 0.01	-54.7	-18.6	-36.1	763 ± 0

^**a **^Numbers in brackets indicate the number of measurements.

**Figure 4:**
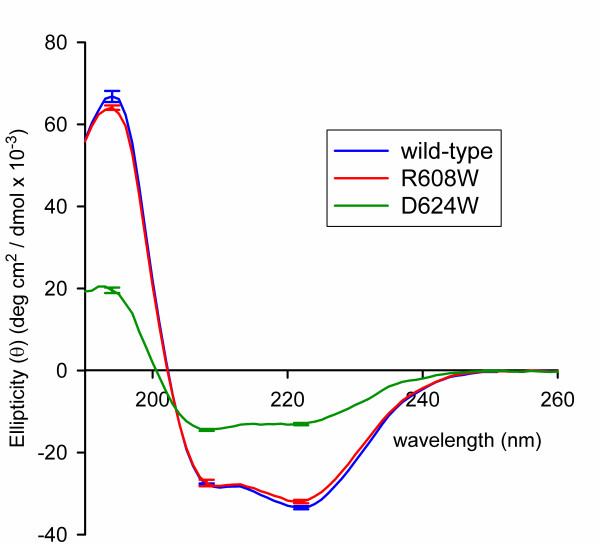
**Circular dichroism analysis of wild-type and mutant Pex5p(C)**. CD spectra of wild-type and the mutant (R608W and D624W) forms of Pex5p(C). While wild-type and R608W show very similar secondary structure content, D624W does not. See text for details.

To determine whether structural alterations induced by the mutations were the cause of the observed binding defects, we subjected the mutants to circular dichroism spectropolarimetry (CD) and static light scattering (SLS) analysis. While both mutants showed similar properties when tested with SLS (Table 2) (i.e. they are found to be monomeric and similarly mondispersed), a difference could be seen with the D624W mutant using CD (Figure 4, Table 3). Wild-type Pex5p(C) is estimated to have 60.3 ± 2.1% α-helical content and R608W 57.0 ± 1.0%, in good agreement with the 60.2 - 64.4% of residues in α-helices (as a proportion the 329 residue fragment consistently used in crystallisation experiments) found within the various chains of apo-human Pex5p(C) in PDB entries 2C0M[4] and 2J9Q[5]. However, D624W carries only 48.7 ± 1.5% α-helix (with a concomitant increase in irregular structure), suggesting that the D624W mutation may alter the folding properties of Pex5p(C). These data show that a specific cargo recognition defect can be seen with the Pex5p R608W mutant when binding full-length SCP2 and that structural impairment was not the cause of this binding defect.

**Table 2:** Summary of static light scattering data

	Pex5p(C) WT	Pex5p(C) R608W	Pex5p(C) D624W
**Molecular weight (Mw)**	32.2^a ^(± 8%)	35.6 (± 25%)	30.3 (± 18%)

**Number weighted mean (Mn)**	31.0 (± 8%)	34.4 (± 20%)	29.9 (± 16%)

**Polydispersity (Mw/Mn)**	1.0 (± 11%)	1.0 (± 32%)	1.0 (± 25%)

**Theoretical Mw**	35.2	35.2	35.3

^a^Values are expressed in kDa

**Table 3:** Summary of secondary structure estimations from CD spectropolarimetry

Pex5p(C) sample	% α-helix ^a^	% β-strand	% other	NRMSD ^c^
**Wild-type**	60.3 ± 1.5 ^b^	7.0 ± 1.0	32.6 ± 0.6	0.012 ± 0.002

**R608W**	57.0 ± 1.0	9.0 ± 1.0	34.0 ± 0.6	0.011 ± 0.002

**D624W**	48.7 ± 1.5	9.0 ± 1.0	42.3 ± 0.6	0.017 ± 0.002

^a^Secondary structure estimates derived from DICHROWEB [17] (see Methods)

^b^Mean and standard deviation over three measurements are given

^c^Normalised root mean square deviation calculated in DICHROWEB [17]

To further clarify the impaired binding between Pex5p(C) mutants and SCP2, we examined the interaction kinetics using the Octet RED96 assay system (Table 4) [6]. Once again, in the absence of the PTS1, no binding could be measured - regardless of whether the presequence in SCP2 or mutations in Pex5p(C) were present. We detected that relative to wild-type Pex5p(C), each mutant displayed a 2.0 - 3.5 fold decreases in association rates (k_on_). Most strongly impaired in k_on _(a 3.5 fold decrease) was the infantile Refsum disease mutant Pex5p(C)S600W [7], a mutation in the 7C-loop (and therefore distant from the ancillary interface) we have previously found not to bind to mSCP2 in ITC experiments and to impair import of SCP2 and catalase *in vivo *[4]. In contrast, all mutants displayed very similar dissociation rates (k_off_) to wild-type Pex5p(C), with the exception of D624W, which had a 2.7 fold more rapid k_off _from mSCP2 compared to wild-type. Thus, in line with our previous data, the ancillary interface residues Arg608 and Asp624 do have roles in receptor recognition and binding but at least in the case of Arg608, play a lesser role in the overall stability of the Pex5p(C)/SCP2 complex. The impaired k_on _and k_off _demonstrated for D624W presumably result from the structural deviation shown above.

**Table 4:** Summary of binding kinetics measured using Octet RED96

Immobilised biotinylated Pex5p(C)	Ligand	Association rate, k_on _ (1/Ms)	Dissociation rate, k_off _ (1/s)
**Wild-type**	mSCP2	2.3 × 10^4 ^(± 2.0 × 10^2^)	5.5 × 10^-5 ^(± 2.0 × 10^-6^)

**Wild-type**	preSCP2	3.5 × 10^4 ^(± 2.8 × 10^2^)	7.9 × 10^-5 ^(± 1.5 × 10^-6^)

**S600W**	mSCP2	6.5 × 10^3 ^(± 3.9 × 10^1^)	6.0 × 10^-5 ^(± 1.7 × 10^-6^)

**S600W**	preSCP2	7.3 × 10^3 ^(± 4.8 × 10^1^)	5.1 × 10^-5 ^(± 1.8 × 10^-6^)

**R608W**	mSCP2	7.2 × 10^3 ^(± 4.8 × 10^1^)	7.3 × 10^-5 ^(± 1.8 × 10^-6^)

**R608W**	preSCP2	9.0 × 10^3 ^(± 6.4 × 10^1^)	5.0 × 10^-5 ^(± 1.9 × 10^-6^)

**D624W**	mSCP2	1.1 × 10^4 ^(± 8.3 × 10^1^)	1.5 × 10^-4 ^(± 2.3 × 10^-6^)

**D624W**	preSCP2	1.3 × 10^4 ^(± 1.1 × 10^2^)	8.6 × 10^-5 ^(± 2.2 × 10^-6^)

### Peroxisomal import of SCP2 in fibroblast complementation assays

**Figure 5:**
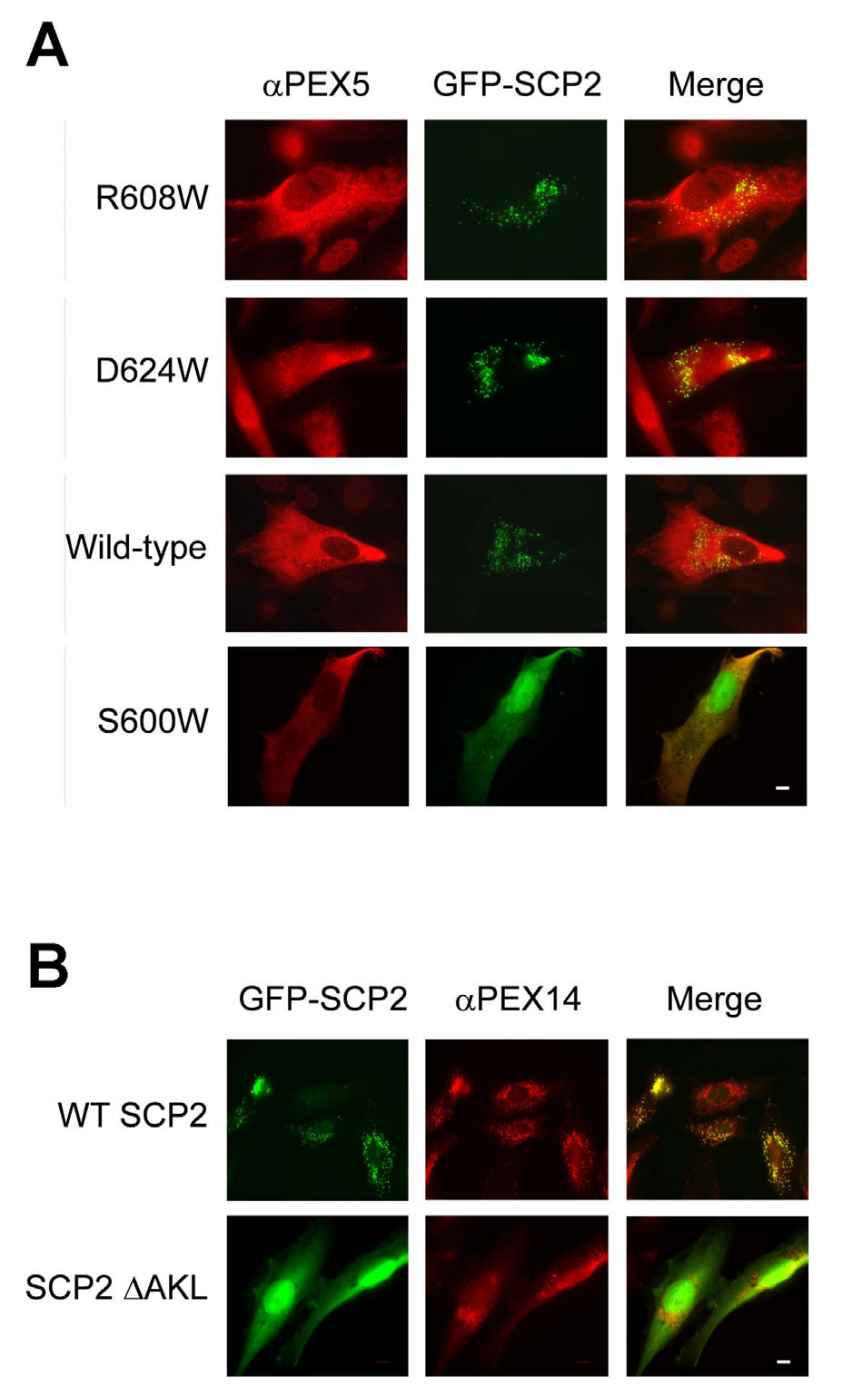
***In vivo *localisation assays**. Subcellular localisation of peroxisomal marker enzymes demonstrate that mutations within the ancillary SCP2 binding site of Pex5p do not impair peroxisomal import. Pex5p-deficient fibroblast cells were co-transfected with a plasmid expressing GFP-SCP2 either with (A) or without its PTS1 sequence (B) and plasmids expressing wild-type (WT) or mutant forms of Pex5p, carrying the point mutations R608W, D624W or S600W. Forty eight hours after transfection GFP-SCP2 was visualised by direct fluorescence (green colour), while Pex5p (A) and Pex14p (B) were detected by immunofluorescence microscopy. Scale bars (in bottom right micrographs) indicate 10 μM.

We have complemented Pex5p impaired fibroblast cultures from Zellweger syndrome patient PBD005 [4] with the long isoform of human Pex5p (Pex5p(L)) and Pex5p(L) mutated at residues Arg608 and Asp624. 12, 24 and 48 hours after transfection, the ability of these complemented cells to import the reporter molecule GFP-mSCP2 into peroxisomes was assayed by immunofluorescence microscopy (Figure 5). We also determined the localisation of Pex5p(L) using immunofluorescence. The cytosolic distribution of Pex5p(L) was not altered by the mutations and all ancillary interface mutants gave rise to a similar punctuate pattern for GFP-mSCP2, characteristic of peroxisomal localisation (Figure 5). Even when the transfected cells were incubated at 40°C instead of 37°C (to challenge the cells with a stress condition) the Pex5p mutants retained their ability to import GFP-SCP2 into peroxisomes (data not shown). In contrast, both negative controls, using Pex5p(L) S600W and wild-type Pex5p(L) with GFP-mSCP2ΔAKL, showed diffuse GFP fluorescence (Figure 5), indicating that in each case GFP-SCP2 is retained in the cytosol. Thus, despite the moderate impairment of *in vitro *binding caused by mutation at the ancillary interface, the same mutations do not impair Pex5p(L) mediated peroxisomal import, at least of SCP2 as an import substrate - although subtle alterations in import kinetics cannot entirely be excluded by our localisation assay. However, the presence of the PTS1 and retention of the capacity of Pex5p(L) to act as a PTS1 binding receptor are essential.

## Discussion

Our data confirm previous findings [4] that the PTS1 of SCP2 is essential for the interaction with the PTS1 receptor Pex5p and for its Pex5p-mediated targeting to the peroxisomal matrix and we additionally characterise the role of the secondary binding site in cargo binding. While mutation of Arg608 in Pex5p(C) impairs the binding affinity, stoichiometry (from two hybrid and ITC experiments) and association rate (from Octet RED96 experiments) with SCP2, its' introduction *in vivo *does not impair the capacity of Pex5p(L) to import SCP2 into peroxisomes, suggesting that the ancillary interface, formed between SCP2 and Pex5p plays only a limited role in the import of SCP2.

The R608W and D624W mutations in Pex5p(C), while not causing an obvious phenotype *in vivo*, do cause some interesting and unexpected behaviour *in vitro*. Firstly, the apparent increase in binding affinity caused by the D624W mutation, an effect that seems to contradict the reduction observed with the R608W mutant. Our CD data indicate that the D624W mutant has altered folding properties, in relation to the wild type and R608W mutant, which may affect the binding to SCP2 in an unexpected way. A reduction in binding affinity can readily be justified, in terms of a loss of contact sites in Pex5p(C). However, the reductions in binding stoichiometries of the mutants are less easily explained. We cannot absolutely rule out the possibility that the Pex5p mutants are able to oligomerise, resulting in a change in the stoichiometry of the interaction. However, our SLS data indicate that both mutants, like the wild type protein, are monomeric in solution, which makes this explanation unlikely. From the Octet RED96 experiments, we see that both mutants exhibit a slower association rate. This, together with the faster dissociation rate observed with the D624W mutant, combined with apparent changes in its' folding properties, could contribute to differences in the stoichiometries.

We are then left with the question as to what the possible function(s) of the secondary interface in PTS1 protein import may be. The capacity for Pex5p to import folded proteins is well known and is likely to represent an essential part of its task since to date no chaperones have been identified within the peroxisomal matrix that could aid in the folding of imported substrates. Therefore, two possible roles for the ancillary interface could be envisaged; increasing the overall stability of the receptor-cargo complex by providing extra contact sites between Pex5p and the PTS1 protein and/or a "quality control" step, allowing Pex5p to check PTS1 proteins for correct folding before importing them into the matrix, since the region in SCP2 recognised by the ancillary interface, unlike the PTS1, is fully folded. However, it may be expected that impairment of either process would result in an inhibition of the import process, which is not the case in our hands. Our data do not rule out the possibility that Pex5p uses this secondary interface with other cargo proteins and indeed, the apparent conservation of the Arg608 residue suggests a potential role in the function of Pex5p. In addition, recent results indicate that other PTS1-containing proteins interact with this same region of Pex5p in a similar way (**K. Fodor, personal communication**). Therefore, factors additional to the ancillary interface may determine the contribution of this binding site to cargo recognition. Recent data indicate that human PTS1 sequences show a range of binding affinities to Pex5p [8]. It is not hard to imagine that proteins with a relatively low binding affinity may require additional contact sites, to improve their targeting, while those with a high binding affinity (such as SCP2) would not. The targeting of GFP-SKL to peroxisomes falls into the last category. GFP (being a non-native import substrate commonly used as a reporter) is not expected to interact with the secondary interface in Pex5p, yet it is still efficiently targeted, indicating that SKL is sufficient to allow targeting in the absence of a secondary interface. Consequently, further data on other PTS1 cargo proteins are required, to provide insights into the role of the ancillary interface in peroxisome translocation.

## Conclusion

The data presented in this study clarify that the mechanism of SCP2 sorting to the peroxisome is absolutely PTS1 dependent but independent of the ancillary interface. It remains to be seen if a broad set of proteins destined for the peroxisome lumen utilise this ancillary binding site, or if the majority rely on a more rugged "PTS1 or nothing" selection, as appears to be the case here.

## Methods

### Materials

Unless otherwise stated, all chemicals were obtained at the highest available purity from Sigma-Aldrich. Restriction enzymes were purchased from New England Biolabs (Ipswich, MA, USA).

### Plasmids and cloning

*E. coli *expression vectors for the production of human Pex5p(C) (spanning residues 315-639), preSCP2 (1-143) and mSCP2 (21-143) complete with N-terminal His_6_-GST fusion, cleavable with tobacco etch virus (TEV) protease have been described previously [4,9]. Primers described in [9] were also used to facilitate insertion of the four SCP2 variants (preSCP2 (1-143), mSCP2 (21-143), preSCP2ΔAKL (1-140) and mSCP2ΔAKL (21-140)) into pPC97 [10] between *Sal*I and *Not*I, to generate fusions with Gal4 DNA binding domain (Gal4DB) for yeast two-hybrid analysis. The fusion of HsPex5p(C) and the Gal4 activation domain (Gal4AD) was made by cloning the *Nco*I-*Not*I fragment from the HsPex5p(C) *E. coli *expression vector into pPC97. Point mutations were introduced into the various Pex5p constructs using the QuikChange™ site directed mutagenesis kit (Stratagene) using either the Gal4AD Pex5p(C) plasmid, the His_6_-GST Pex5p(C) plasmid, or the pcDNA3 derived expression vector pGD106 [11] as templates. Details of the primers used can be seen in Table 5. The Pex5p S600W mutant has been described previously [4]. All constructs were confirmed by DNA sequencing.

**Table 5:** PCR primers used in this study

Primer name	5'-3' sequence
**HsPex5p: R608Wf**	CATCTGGAGCACCCTGTGGTTGGCATTGTCTATGTTAGG
**HsPex5p: R608Wr**	CCTAACATAGACAATGCCAACCACAGGGTGCTCCAGATG
**HsPex5p: D624Wf**	CCTATGGGGCAGCCTGGGCGCGGGATCTGTC
**HsPex5p: D624Wr**	GACAGATCCCGCGCCCAGGCTGCCCCATAGG

### Strains and culture conditions

Recombinant production of human Pex5p(C), preSCP2 and mSCP2 in *E. coli *has been described before [4,9]. The yeast strain *S. cerevisiae *PCY2 (*MATΔ, Δgal4, Δgal80, URA3::GAL1-lacZ, lys2-801, his3-Δ200, trp1-Δ63, leu2, ade2-101*) was used for two-hybrid analysis. Yeast transformations were performed as described in [12]. Transformants were grown on minimal medium containing 0.67% yeast nitrogen base (Difco), 2% glucose and amino acids (20 μg/ml) as required.

### Yeast two-hybrid analysis and Western blotting

GAL4 based yeast two hybrid analysis was conducted as described in [10] with β-galactosidase enzyme activity determination being performed as in [13]. Samples of lysates taken from the β-galactosidase assay were analysed by Western blotting for human Pex5p expression levels. Proteins were separated on a SDS-polyacrylamide gel and blotted onto a nitrocellulose membrane using a semi-dry system. Antibodies used were directed against *S. cerevisiae *hexokinase (generous gift of H. van der Spek, FNWI, Amsterdam, The Netherlands) and human Pex5p described in [14] Antibody binding was detected with secondary antibodies labeled with IRDye 680 infrared dye on an Odyssey imager (LI-COR Biosciences).

### Protein preparation and biotinylation

Preparation of Pex5p(C), preSCP2 and mSCP2 has been described previously [4,9]. Mutant Pex5p(C) proteins were prepared in a similar way as described in [4]. Purity was monitored by SDS-PAGE and mass spectrometry. For concentration determination, proteins were denatured in 8 M urea and the A_280 nm _was measured. Extinction coefficients were calculated using the method of Gill and von Hippel [15]. Pex5p(C) wild-type and mutants were biotinylated using the EZ-Link^® ^Sulfo-NHS-Biotinylation Kit (Pierce) as described by the manufacturer. Using the 4'-hydroxyazobenzene-2-carboxylic acid/avidin method, between 1 and 2 moles of biotin were found incorporated with 1 mole of Pex5p(C).

### Isothermal titration microcalorimetry (ITC)

ITC was performed as described in [4]. Measurements were conducted using a MicroCal VP-ITC using either wild-type or mutant (R608W or D624W) forms of Pex5p(C) titrated with either mSCP2 or a peptide derived from the C-terminus of SCP2 [4]. The peptide, PGNAKL, was synthesised by Sigma-Aldrich at > 95% purity.

### Circular dichroism spectropolarimetry

Circular dichroism spectropolarimetry (CD) was performed as described in [16] with Pex5p(C) at a concentration of 2 - 5 μM in 10 mM Potassium Phosphate buffer pH 7.4. Each spectrum presented is the average of three measurements. Standard deviations in ellipticity are shown with error bars. Spectra were background subtracted and helical content estimated via the DICHROWEB interface [17], using CDSSTR [18] with the SP175 basis set of spectra.

### Static light scattering

The procedure used for static light scattering (SLS) analysis is described in [19].

### Octet RED96 kinetic assays

To assess the binding kinetics of Pex5p(C) wild-type and point mutants to the four SCP2 variants, the Octet RED96 instrument (ForteBio) was used [6]. All steps were carried out in phosphate-buffered saline (PBS) at a rotation rate of 1000 rpm. Streptavidin conjugated sensors were hydrated for 10 minutes prior to use. The biotinylated sample proteins were immobilised onto the sensor at a concentration of 20 μg/mL. The experiment proceeded as follows; baseline - 60 seconds, biotinylated-sample loading - 300 seconds, baseline 2 - 60 seconds, association - 1800 seconds and dissociation - 1800 seconds. Data were acquired and assessed using the custom ForteBio software Data Acquisition v6.2 and Data Analysis v6.3. The data were processed with the Savitzky-Golay filter prior to analysis. Lines of best fit were generated locally based on a 1:1 model to determine the relative binding kinetics.

### In vivo peroxisome import assays

Culturing and transfection of the Pex5p deficient human skin fibroblast cells for Zellweger syndrome patient PBD005 was performed as described in [4]. 12, 24 and 48 hours after transfection, cells were fixed onto cover glasses with 3% formaldehyde in PBS, permeablised with 1% Triton X-100 in PBS and subjected to immunofluorescence microscopy. To test for temperature-sensitive import defects, the temperature of cell culture was shifted from 37 to 40°C for 2 days. Polyclonal rabbit antibodies directed against human Pex14p are described in [20]. Polyclonal rabbit anti-Pex5p antibodies were raised against recombinant His_6_-tagged Pex5p expressed in *E. coli *and purified as described previously [14]. Secondary antibodies were conjugated with Alexa Fluor 594 (Invitrogen). Samples were also inspected for GFP fluorescence. All micrographs were recorded on a Zeiss Axioplan 2 microscope with a Zeiss Plan-Apochromat 63x/1.4 oil objective and an Axiocam MR digital camera and were processed with AxioVision 4.2 software (Zeiss).

## Abbreviations

**CD**: Circular dichroism spectropolarimetry; **GFP**: green fluorescent protein; **ITC**: isothermal titration calorimetry; **PBS**: phosphate buffered saline; **PTS**: peroxisomal targeting signal; **SCP2**: sterol carrier protein 2; **SLS**: Static light scattering; **TPR**: tetratricopeptide repeat.

## Authors' contributions

Experimental data were collected and analysed by CPW, NS, CAT, MvdB, SDvH, CSB, WS and WAS. The study was conceived and designed by CPW, RE, WS, MW and WAS. CPW, NS, CAT, RE, CSB, BD, WS, MW and WAS interpreted the data and prepared the manuscript. All authors read and approved the final manuscript.
